# Pleomorphic Adenoma of the Nasal Septum: A Case Report

**DOI:** 10.7759/cureus.24400

**Published:** 2022-04-22

**Authors:** Nur Jannah Isdeehar Salahuddin, Mohd Eksan Sairin

**Affiliations:** 1 Otolaryngology - Head and Neck Surgery, Hospital Miri, Miri, MYS

**Keywords:** benign tumour, salivary glands, pleomorphic adenoma, nasal septum, nasal cavity

## Abstract

Pleomorphic adenoma is the most common benign tumor of major and minor salivary glands. It rarely originates from the nasal cavity. It is important to identify the symptoms and characteristics of the disease so that early diagnosis can be made and appropriate treatment can be offered as it has a risk of malignant transformation. We are reporting a case of a 22-year-old female with a long duration of unilateral nasal obstruction. Rigid nasoendoscopy showed a mass at the left nasal septum. Preoperative imaging was performed followed by endoscopic resection of the mass. The pathological diagnosis was pleomorphic adenoma. Post-operatively, appropriate examination with nasoendoscopy and long-term follow-up is essential to avoid local recurrence.

## Introduction

Salivary gland tumors constitute 3% of head and neck tumors, and the most common benign tumor is pleomorphic adenoma. In 70% cases, pleomorphic adenoma arises from the major salivary gland, and around 10% originate from the minor salivary gland [1-3]. It is a mixed tumor type that is composed of two types of cells: epithelial and myoepithelial structures [3,4]. The epithelial component can undergo malignant transformation in isolation or with stroma, which can be an aggressive malignant tumor with a five-year mortality rate of 30-50% [3,5].

Pleomorphic adenoma of the minor salivary glands can be found at locations where the minor salivary glands exist, such as the neck, ear, mediastinum, external nose, cheek, and nasal cavity [2,4,6,7]. Lesion detected in the nasal cavity is rare, and for most of the cases, the tumor originates from the mucosa of the nasal septum even though the seromucosal glands are mainly at the lateral nasal wall [2,3,7].

We report the case of a patient who presented with left nasal block and rhinorrhea for two years. Imaging and biopsy help in confirming the disease and hence proper treatment can be started.

## Case presentation

A 22-year-old female with underlying childhood bronchial asthma presented to the Otorhinolaryngology Outpatient Department with the complaint of left nasal blockage and rhinorrhea for two years. Otherwise, there were no other nasal symptoms such as epistaxis, foul smelly discharge, or facial pain and swelling. No cervical lymph node enlargement was noted. She also denied any constitutional symptoms, ear symptoms, throat symptoms, or history of trauma to the nose. There were no significant inputs regarding the past medical and surgical history.

**Figure 1:**
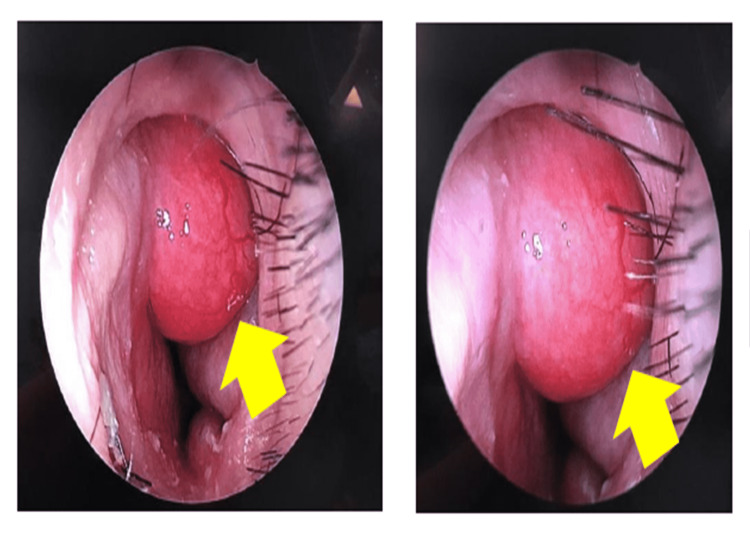
Left septal mass (yellow arrow)

Rigid nasoendoscopy was performed, which showed a globular, smooth surface mass with prominent vessels originating from the left side of the septum (Figure 1). It was firm in consistency and not bleeding upon touch. Other examinations showed no significant findings.

**Figure 2:**
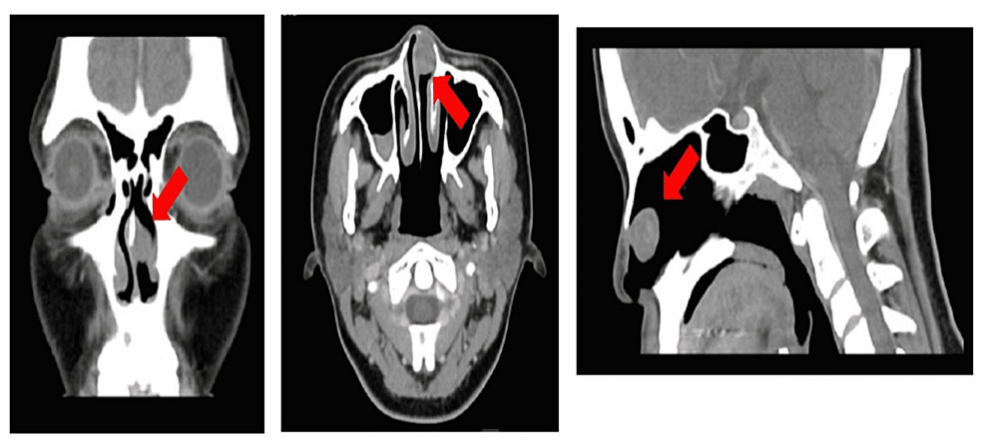
Well defined enhancing soft tissue lesion (red arrow).

Computed tomography (CT) of the paranasal sinus showed a well-defined heterogeneously enhancing soft tissue lesion at the anterior aspect of the left nasal cavity, measuring 1.6 x 1.3 x 1.7 cm, and limited in the nasal cavity. The mass causes a contour bulge toward the nasal septum, causing slight right deviation of the nasal septum. It had no extension into the subcutaneous tissue, toward the right nasal cavity, or next to the anterior aspect of the left inferior turbinate with no clear plane demarcation in between (Figure 2). There is also associated remodelling with cortical thinning of left-sided nasal bone. Bilateral parotid and submandibular glands are normal.

**Figure 3:**
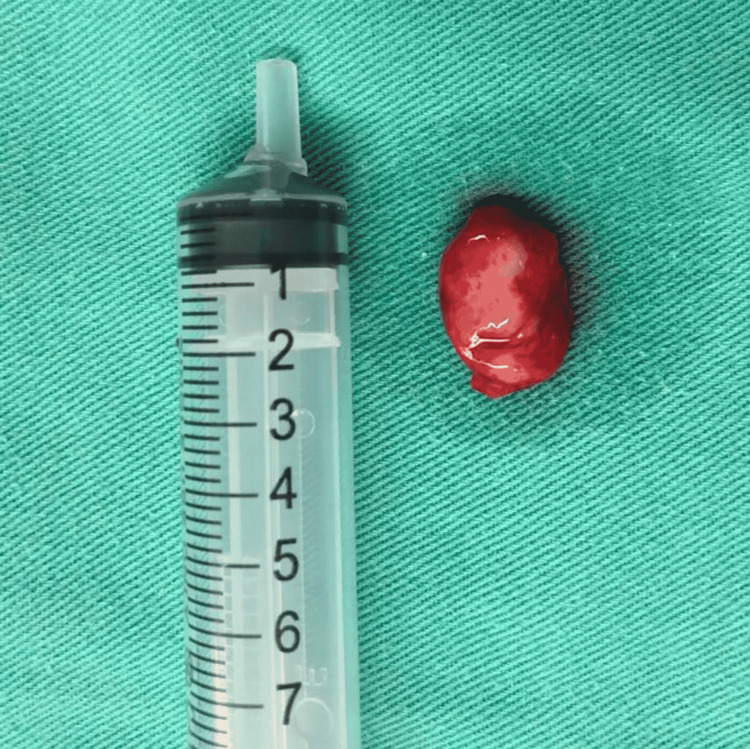
Left septal mass post-excision.

**Figure 4:**
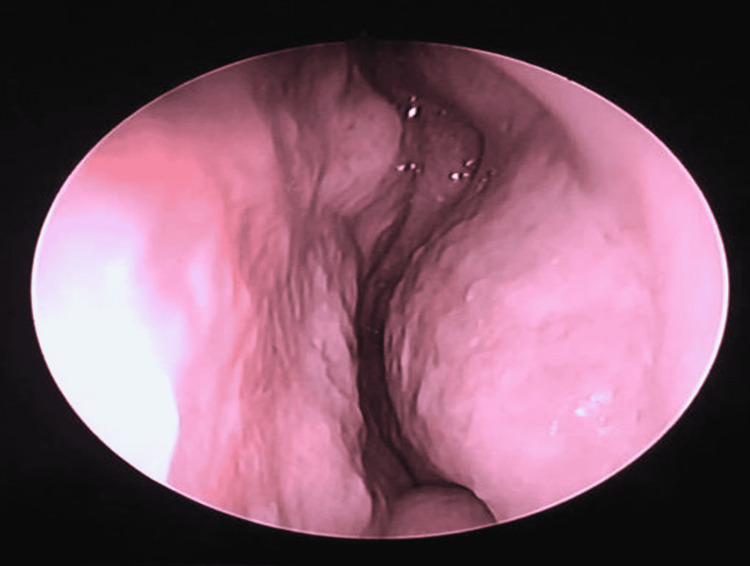
No evidence of recurrence post-excision.

Endoscopic excision of the left septal mass was performed. A vertical incision was made in the left nasal septal mucosa anterior to the mass, and the mucoperichondrium was elevated. Left side of the nasal septum was not involved, and there was no evidence of cartilage invasion. The mass was found to arise from the anterior part of the left septal mucosa, measuring 1 x 1 cm (Figure 3), and excision was performed. Left nasal splint and anterior nasal packing was inserted over the left nasal cavity. Pathology examination found fragments of grey brownish tissue, with the largest measuring 10 x 15 x 5 mm. The histopathology examination revealed it to be pleomorphic adenoma composed of epithelial and myoepithelial cells with predominant myoepithelial component. The post-operative period was uneventful, the left nasal packing was removed on the next day, and the patient was discharged from the hospital. She was followed up two weeks later to review and remove the nasal splint and then followed up monthly and subsequently every four months. After one year of surgical intervention, there was no sign of recurrence of growth noted during rigid nasoendoscopic examination on each follow-up (Figure 4).

## Discussion

Pleomorphic adenoma is the most common benign salivary gland tumor and constitutes around 65% of all salivary gland neoplasms. It can arise from parotid glands, submandibular glands, or other minor salivary glands. It also can occur in the respiratory tract via the minor salivary glands, which is very rare [2,3,7]. In the upper respiratory tract, usually it is found in the nasal cavities followed by the maxillary sinus and nasopharynx. Approximately 80% pleomorphic adenomas of the nasal cavity originate from the nasal septum, while others originate from the lateral nasal wall or turbinate [1,7].

Intranasal mass has many differential diagnoses such as benign lesions (inverted papilloma, squamous papilloma, pleomorphic adenoma, schwannoma, chondroma, encephalocele) or malignant lesions (squamous cell carcinoma, malignant melanoma, lymphoma, cartilaginous tumors) [1,7]. Hence, investigation is needed to diagnose the disease so that treatment can be offered accordingly. Intranasal pleomorphic adenoma can occur at the age of 30 to 60 years and is predominantly more common in females [3,7]. The rate for malignancy transformation is 2.5-10%.

Patients can have painless, unilateral nasal blockage, epistaxis, sinusitis, or mass within the nasal cavity. Other nasal symptoms such as nasal swelling, anosmia, epiphora, and mucopurulent rhinorrhea may also be present [2,3,6]. Rhinoscopy or rigid nasoendoscopy will show a unilateral, smooth surface, pale grayish whitish mass [4,7]. As for our case, she had left nasal blockage with rhinorrhea, and rigid nasoendoscopy showed unilateral, globular, and smooth surface mass over the left septum. Imaging studies such as CT scan and magnetic resonance imaging (MRI) are useful in determining the origin of intranasal mass when it is smaller in size and accompanied by neighboring bony changes. CT scan may show a distinct, lobulated mass displacing the nasal septum [4]. As for our patient, her CT scan fit the criteria as it showed a well-defined mass, which also caused a slight right-deviated nasal septum.

Pleomorphic adenoma is a slow-growing tumor, but it has a high rate of recurrence of approximately 50% for parotid gland tumors and low recurrence rate of 10% for intranasal tumors [6]. Histopathology examination shows manifestation of both epithelial and mesenchymal components. It is usually encapsulated in the major salivary gland and nonencapsulated in the minor salivary gland [7]. The literature showed that pleomorphic adenoma also has a risk of malignant transformation; hence, excision is justified in nearly all cases. Nasal septal pleomorphic adenoma is treated by total excision with clear, wide surgical margin, and the depth is based on radiological and intraoperative findings. This is important to avoid disease recurrence [1-3]. Options for removal of tumor vary depending on the size and location of the tumor, such as endoscopic resection, intra-nasal excision lateral rhinotomy, and midfacial degloving [1,2,6,7]. Nasal endoscopic resection was performed in our patient as the mass was limited to the anterior aspect of the nasal cavity.

After treatment, long-term follow-up is needed to monitor any evidence of local recurrence [2]. Some studies reported that imaging (CT scan or MRI) was repeated during follow-up [7]. However, in our patient, as her symptoms resolved post-operatively and during each follow-up, rigid nasoendoscopic examination had no significant finding and hence no imaging was repeated.

## Conclusions

In conclusion, when a patient presented with unilateral mass in the nasal cavity, pleomorphic adenoma should be one of the differential diagnoses, although it is not common. Early detection is necessary so that complete excision can be performed at an earlier stage to avoid recurrence. Long-term follow-up is required due to the risk of malignant transformation.
